# The Role of Innate Immunity in Natural Elite Controllers of HIV-1 Infection

**DOI:** 10.3389/fimmu.2022.780922

**Published:** 2022-02-08

**Authors:** Yuting Shi, Jinming Su, Rongfeng Chen, Wudi Wei, Zongxiang Yuan, Xiu Chen, Xinwei Wang, Hao Liang, Li Ye, Junjun Jiang

**Affiliations:** ^1^ Guangxi Key Laboratory of AIDS Prevention and Treatment, School of Public Health, Guangxi Medical University, Nanning, China; ^2^ Joint Laboratory for Emerging Infectious Diseases in China (Guangxi)-ASEAN, Life Sciences Institute, Guangxi Medical University, Nanning, China

**Keywords:** HIV/AIDS, elite controllers, long-term nonprogressors, innate immunity, PRR

## Abstract

The natural process of human immunodeficiency virus type 1(HIV-1) infection is characterized by high viral load, immune cell exhaustion, and immunodeficiency, which eventually leads to the stage of acquired immunodeficiency syndrome (AIDS) and opportunistic infections. Rapidly progressing HIV-1 individuals often die of AIDS several years after infection without treatment. The promotion of ART greatly prolongs the survival time of HIV-infected persons. However, some patients have incomplete immune function reconstruction after ART due to latent storage of HIV-infected cells. Therefore, how to achieve a functional cure has always been the focus and hot spot of global AIDS research. Fortunately, the emergence of ECs/LTNPs who can control virus replication naturally has ignited new hope for realizing a functional cure for AIDS. Recently, a special category of infected individuals has attracted attention that can delay the progression of the disease more rigorously than the natural progression of HIV-1 infection described above. These patients are characterized by years of HIV-1 infection, long-term asymptomatic status, and normal CD4+T cell count without ART, classified as HIV-infected long-term nonprogressors (LTNPs) and elite controllers (ECs). Numerous studies have shown that the host and virus jointly determine the progression of HIV-1 infection, in which the level of innate immunity activation plays an important role. As the first line of defense against pathogen invasion, innate immunity is also a bridge to induce adaptive immunity. Compared with natural progressors, innate immunity plays an antiviral role in HIV-1 infection by inducing or activating many innate immune-related factors in the natural ECs. Learning the regulation of ECs immunity, especially the innate immunity in different characteristics, and thus studying the mechanism of the control of disease progression naturally, will contribute to the realization of the functional cure of AIDS. Therefore, this review will explore the relationship between innate immunity and disease progression in ECs of HIV-1 infection from the aspects of innate immune cells, signaling pathways, cytokines, which is helpful to provide new targets and theoretical references for the functional cure, prevention and control of AIDS, and development of a vaccine.

## Introduction

HIV, the human immunodeficiency virus, can lead to a progressive loss of immune function, eventually leading to acquired immunodeficiency syndrome (AIDS). AIDS is a major public health problem globally, posing a severe threat to human health and social stability. There is still no effective vaccine or cure. HIV can be divided into HIV-1 and HIV-2. The global prevalence of different subtypes varies greatly, with the HIV-1 epidemic widespread worldwide (1, 2). According to the World Health Organization, 37,700,000 people were living with HIV globally by the end of 2020, 1,500,000 people were newly infected, and 680,000 patients died from HIV-related causes in 2020. HIV-1 infection is a dynamic process with different rates of disease progression. Rapidly progressing HIV-1 individuals often die of AIDS several years after infection without treatment. The promotion of ART greatly prolongs the survival time and reduces the mortality rate of HIV-infected persons (3). However, HIV-infected patients cannot eradicate the virus even with lifelong ART because of latent viral reservoir (LVR) (4). Therefore, researchers proposed the concept of functional AIDS cure, that is, long-term control of HIV replication after discontinuation of treatment to maintain the normal number and function of CD4+T cells. Although patients carry the virus, their viral load is lower than the detection limit, and there are no typical pathological features. However, how to achieve a functional cure has always been the focus and hot spot of global AIDS research. So far, functional healing has been achieved in ECs/LTNPs, who still have replicating viruses and a low level of the latent reservoir, but the body can control the virus spontaneously and effectively below the detection limit without ART. By comparing those with the natural progression of HIV infection, HIV-1-infected natural elite controllers are a special group of HIV-infected patients, including LTNPs and ECs, who bring a new hope and breakthrough point for a functional cure. LTNPs refer to patients who have been infected with HIV-1 for 10 years or longer, although they have never received antiretroviral therapy (ART), and maintain a CD4+ T lymphocyte count at a normal level (>500/µl). The immune system maintains a long-term stable state in these patients (5). LNTPs account for about 5% of all people infected with HIV-1 (6); LTNPs with a viral load < 50 copies/ml are known as ECs, accounting for about 0.1 - 1%.

A large number of studies have shown that the host and virus jointly determine disease progression after HIV-1 infection, in which the activation level of innate immunity plays an important role (7, 8). At present, there have been many reports on the adaptive immune mechanism of natural controllers of HIV-1 infection, but relatively little is known about the role of innate immunity. As the first line of defense against the invasion of pathogens, innate immunity is also a bridge to induced adaptive immunity. Pattern recognition receptors recognize HIV infection, and then a series of immune cell responses are recruited to induce or activate many innate immune-related factors to play an antiviral role (9). These innate immune components (10) include skin mucosal epithelial cells, phagocytes, NK cells, as well as a series of soluble factors, such as cytokines, chemokine, and small molecular substances, such as complement and mannose-binding lectin. Together, these elements constitute a rapid response system against infection and play a role in preventing the spread of infection. In addition, antigen-presenting cells, such as dendritic cells and monocytes, exert a certain role in innate and adaptive immunity. Therefore, a further understanding of the mechanisms of innate immunity in ECs of HIV infection is helpful to provide new targets and theoretical references for the functional cure, prevention, and control of AIDS and the development of vaccines. This review will summarise the mechanisms observed in natural progressors, LTNPs, and ECs of HIV infection concerning innate immune cells, pattern recognition receptors, signaling pathways, soluble factors, and then discuss the relationship between innate immunity and disease progression following HIV infection, to provide a reference for further studies on HIV infection in natural ECs.

## Innate Immune Cells Involved in the Natural Control of HIV Infection

**Figure 1:**
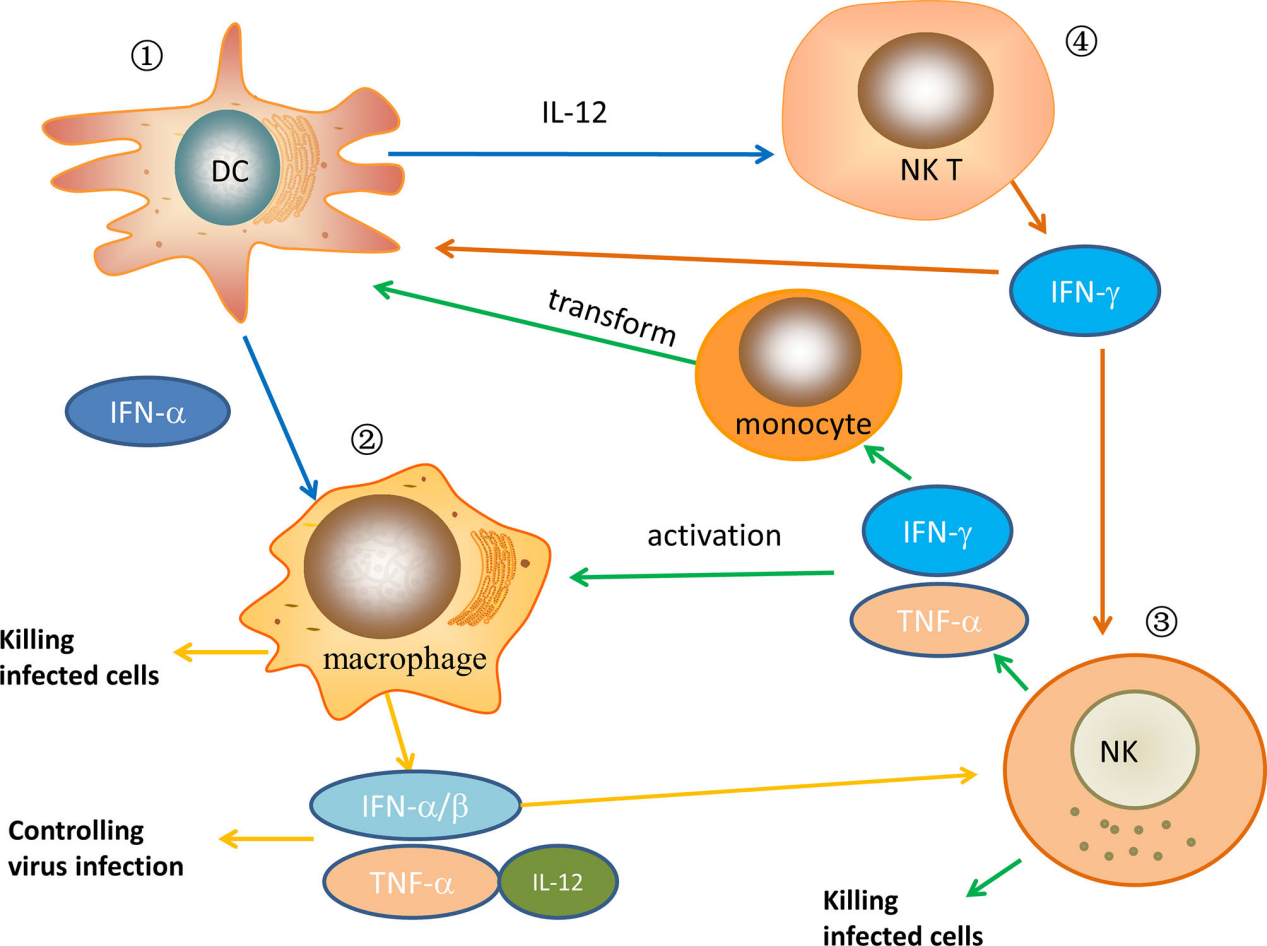
The relationship of some major innate immune cells after HIV infection in ECs/LTNPs. ① In ECs/LTNPs, DCs are activated, the number of cells increases, and cytokines, such as IFN-α and IL-12, are secreted to play the role of antiviral infection. Meanwhile, these cytokines can activate macrophages and NKT cells. ② When macrophages are activated in ECs/LTNPs, the number of macrophages increases, and they have strong phagocytosis to infected cells. At the same time, the secretion of inflammatory cytokines such as IFN-α, IFN-β, TNF-α, and IL-12 increases, which control virus replication and activate NK cells. ③ When NK cells are activated in ECs/LTNPs, the number of cells increases and can directly kill infected cells. In the meantime, its cell activity increased, secreting IFN-γ, TNF-α, which further activates macrophage and induces monocytes to transform into DCs. ④ After NKT cells are activated in ECs/LTNPs, the secretion of IL-2, IFN-γ, and other cytokines increases, which further activate DCs and NK cells. In HIV-infected ECs or LTNPs, these immune cells jointly play an antiviral infection role.

Innate immune cells play a critical role at the onset of HIV infection and remain active until the final events that characterize AIDS, including monocytes/macrophages, dendritic cells, NK cells, NKT cells, γδ T cells, B1 cells, mast cells, various granulocytes (11). Among these cells, in the HIV-infected ECs/LTNPs population, DCs, NK cells, macrophages cells, and NKT cells play an important and irreplaceable role in innate immunity, while other innate immune cells are less effective or even not involved in research. As powerful antigen-presenting cells, dendritic cells play a crucial role in the host’s early response to viruses. Not only can they modulate the host’s rapid response to pathogens, but they also stimulate adaptive immunity (12). Regarding NK cells, there is considerable evidence that NK cells play a key role in the control of chronic HIV infection through antibody-mediated cytotoxicity or secretion of soluble cytokines. The activation of NK cells is partly driven by pro-inflammatory cytokines secreted by dendritic cells and monocytes, including IL-15 and IFN-α. Besides, macrophages are the main effector cells involved in the late innate immune response. They actively cooperate with the recruitment of inflammatory cells by secreting many cytokines, such as IL-1 and TNF-α (13), and have a vital effect on viral immune responses. NKT cells involve complex interactions among various immune cells and exert a strong immunomodulatory effect. Currently, many studies have shown that these innate immune cells are very important to affect the course of the disease after HIV invades the human body in LTNPs/ECs. Thus, in this section, we mainly discuss the cellular immune function of these innate immune cells in the ECs or LTNPs, and we will briefly discuss or not discuss other innate immune cells. The relationship of some major innate immune cells after HIV infection in ECs/LTNPs is depicted in **Figure 1**.

### Relationship Between Dendritic Cells and the Progression of HIV Infection

Dendritic cells are the most powerful type of antigen-presenting cells (APCs) and play a crucial role in the entire process of HIV infection. According to their functions, they can be divided into myeloid dendritic cells (mDCs) and plasmacytoid dendritic cells (pDCs). Both mDCs and pDCs can be infected with HIV, but their susceptibility to different strains of HIV varies.

mDCs are the main antigen-presenting cells in the human body, stimulating and activating T lymphocytes in the body. A study (14) analyzed a group of ECs and found that, compared with progressive HIV infected patients or healthy people without HIV infection, EC subjects had circulating myeloid dendritic cells with significantly increased antigen presentation characteristics and significantly reduced ability to secrete pro-inflammatory cytokines. This unique functional characteristic is associated with a special expression pattern of leukocytes bearing immunoglobulin-like receptors (LILR) on the surface and highly selective up-regulation of LILRB1 and LILRB3. When these two receptors were blocked by monoclonal antibodies or short interfering RNA (siRNA), dendritic cells’ specific antigen presentation characteristics disappeared, suggesting that these molecules performed a significant function in regulating dendritic cells. The results demonstrated that mDCs were of great significance in the immune protection against HIV infection in the special group of ECs. Nevertheless, pDCs also play a role in producing type I interferons (IFN) responses to viral infection. Quite a few studies have revealed that the number of pDCs in the blood usually decreases during chronic HIV infection and is closely related to the progression of HIV infection. Some researchers (15) have compared the ECs with other HIV infection groups and found that the pDCs of the ECs had a greater ability to reduce the production of HIV and induce T cell apoptosis *in vitro*, while the pDCs from patients with viral disease had almost no response without previous toll-like receptor 9 (TLR-9) stimulation, suggesting that pDCs in ECs may be involved in the control of HIV; however, the specific mechanism is still unclear. However, studies have shown that, during the progressive infection of HIV, compared to a seronegative control group, pDCs in the peripheral blood declined and were significantly reduced in the EC group, indicating that pDCs are highly correlated with the progression of HIV infection (16).

The progression of HIV infection may be related to the number and phenotypic function of DCs. A longitudinal study showed that, compared to HIV-1 ECs, in the typical progression of HIV-1 infection, the number and phenotype of DCs changed as the disease progressed, suggesting that DCs are correlated with the progression of HIV infection (17). Furthermore, it has been demonstrated (18) that DCs in HIV-infected ECs can enhance and expand the ability to stimulate HIV-specific CD8+ T cell responses by improving the intrinsic immune recognition of HIV-infected cells, implying that type I interferon secreted by dendritic cells plays an important role in inducing effective HIV-specific CD8+ T cell immunity and may contribute to inducing HIV-specific functional T cell immunity and influencing the disease process. Meanwhile, some studies have discovered that after HIV exposure, ECs had a high functional antiviral DC status, and some of their abundances were associated with higher CD4+ T cell counts and lower viral load, effectively initiating the multifunctional T cell response *in vitro* and controlling disease progression (19).

Furthermore, some studies have done a large-scale longitudinal cytometric analysis of blood samples of early HIV-infected persons before and after receiving effective combined antiretroviral therapy for one year. The results showed that the plasma HIV RNA correlated with the loss of cDC and pDC subsets, showing high expression of the LILR immunity receptor. HIV-controlled patients also had more specific subtypes of CD1c+ cDCs in their blood, characterized by increased co-expression of CD32b inhibitory receptors and HLA-DR antigen-presenting molecules. These data suggest that dendritic cells are dysfunctional after HIV infection, which might help set up the balance of viral persistence (20), thus affecting the course of infection.

### The Role of Natural Killer Cells in Controlling HIV Infection

Natural killer cells (NK) are part of the innate immune system and are crucial in controlling viral infection. NK cells can be divided into two different subpopulations according to the expression of CD56 and CD16 (21). CD56 dim NK cells have high cytotoxicity by eradicating cell targets for tumor transformation or viral infection through cytotoxic effector factors or producing IFN-γ after activation. In contrast, CD56 bright NK cells have low cytotoxic capacity but produce high levels of cytokines, which mainly perform immune regulatory functions. CD16 is a strong activator of NK cell function and controls virus infection by mediating antibody-dependent cellular cytotoxicity (ADCC) (22). Recent evidence suggests that CD16 may play a role in the pathogenesis of HIV. The frequency, phenotype, and function of NK cells are highly affected by the HIV-1 virus, and pathological changes occur as the disease progresses. A study (23) showed that NK cell activity was within the normal range in LTNPs, but decreased in patients with disease progression, suggesting that NK cell activity is an important factor in controlling HIV-infected disease progression. However, another study (24) found that the CD56+ NK cell activity level of LTNPs was higher than that of patients with HIV progression. Meanwhile, a few studies revealed that an increase in the percentage of IFN-γ(+)CD107a(-) NK cells in LTNPs compared with the typical progressors, indicating that enhanced NK cell function might contribute to the control of HIV infection and reduced IFN-γ secretion might play an important role in the delay of disease progression (25). Furthermore, an analysis of different lymphocyte subsets in a group of LTNPs HIV-infected children over 8 years of age showed that, compared with healthy controls, NK cells in LTNPs and antiretroviral therapy group were significantly lower (26), suggesting that age may affect NK cells as well.

Killer immunoglobulin-like receptors (KIRs) are important recognition receptors expressed on the surface of NK cells that regulate NK cell inhibition and/or activation after interaction with human leukocyte antigen (HLA) class I ligands. Different KIR genes may influence the prognosis of many different diseases. Many studies have suggested that the anti-NKP44L monoclonal antibody reduces the lytic activity of autologous CD4+ T cells after treatment with LTNPs and HIV progressors, indicating that NKp44L plays a key role in LTNPs and HIV progressors (27). A further study found that, in LTNPs, the level of NKp46 expression was normal but brief (2 days), which of NKp30 was lower than that of healthy donors, but the expression level of NKp44, which is an effective inducer of activated NK cells, was not induced. In addition, NKG2D expression increased on NK cells in the peripheral blood of ECs/LTNPs, HLA-DR was insignificant up-regulation, and mature NKG2A-CD57+ killer cell Ig-like receptor CD85j+ phenotype showed the lytic function of killing dendritic cells. Therefore, NK cells could maintain unchanged function in ECs/LTNPs, while NKP44-induced deficiency might be related to the maintenance of CD4+ T cells (28). On the other hand, in terms of gene levels, the frequency of KIR3DS1/L1 heterozygotes carrying the HLAH-BW4-80i gene was much higher in LTNPs than in the typical progression (TP) group. Meanwhile, the expression level of KIR3DS1 mRNA was higher in the LTNP group, while that of KIR3DL1 mRNA was higher in the TP group (29), indicating that different KIR-HLA genotypes and different levels of transcription are associated with HIV disease progression.

Since bacterial or viral DNA contains some short nucleotide sequences with immune activity, the characteristic structure is non-methylated cytosine guanine dinucleotide (CpG). The human immune system recognizes pathogenic microorganisms by recognizing the characteristic CpG-DNA structure, thus producing a protective immune response against pathogens. Several lines of evidence have suggested that mammalian cells rely on TLR9 to distinguish themselves from pathogen DNA, namely CpG DNA indicating microorganisms, to induce or influence the occurrence of innate immune responses. Some studies have shown that the production of IFN-γ by NK cells was significantly reduced in HIV-infected progressors in response to CpG type A oligonucleotides, compared with LTNPs, and healthy individuals’ defect was negatively correlated with the frequency of mDCs. In addition, LTNPs peripheral blood monocytes and peripheral blood monocytes from healthy HIV-negative donors secreted large amounts of IL-12 and responded to CpG in un-stimulated cultures, while cells from HIV progressors showed impaired responses and low levels of spontaneous secretion. The addition of an IL-12 monoclonal antibody reduced the response to CpG in a dose-dependent manner. These results indicate that impaired NK cell responses to CpG in HIV progressors largely depend on a reduction in IL-12 (30). In addition, in a group of female HIV-infected patients, the frequencies of dendritic cells and CD56+ NK subsets were similar in LTNPs and progressive HIV patients, and the typical CD56+CD16+ NK subsets and CD56+ NK subsets were significantly higher than those in LTNPs, indicating that an increase in the number of NK cells and T cells was associated with the increase in the amount of HIV shedding in the cervix of HIV-infected women, suggesting that NK cells might play different roles in controlling disease progression in different genders (31).

### Macrophages in HIV Infection

Macrophages originating from monocytes play important roles in innate and adaptive immune responses. The ability of HIV to infect macrophages is dependent on their activation status and the amount of expression of inflammatory markers associated with the course of AIDS illness, although the specific processes remain unclear.

Monocyte chemotactic protein-1 (MCP-1) and macrophage stimulating protein (MSP) have been recently identified as two important regulatory factors of macrophages. Some studies (32) have examined the levels of macrophage inflammatory protein (MIP), IFN-γ, and IL-2 in a group of ECs and discovered that ECs exhibited more strong and complicated HIV-specific T cell responses in the rectal mucosa, suggesting that ECs may play an essential role in immune surveillance. Simultaneously, MIP-1, IFN-induced protein 10 (IP-10), MCP-1, and transforming growth factor levels in ECs dropped (33). Another study (34) showed that the medial levels of IL-4, IFN-γ, and granulocyte-macrophage stimulating factor in ECs were twice as high in nonprogressors or individuals who were not infected with HIV. These findings imply that HIV ECs keep viral replication low by secreting several regulatory factors from macrophages (35). Nonetheless, some researchers have discovered that certain ECs had low MIP-1, IFN-γ, and IP-10 levels, as well as low CD4+ T cell numbers. As a result, even modest levels of HIV replication in LTNPs may be associated with a steady decline in CD4+T cell counts (36). Increased IL-2 and MIP-1 levels have been shown in studies to mediate the immunological superiority of Gag T cells and CD8+T cells in the early stages of HIV infection, maintain CD4+T cells, and regulate disease progression (37). These data suggest that macrophage regulatory factors and inflammatory proteins are associated with the progression of AIDS.

On the other hand, macrophage lineage cells with persistent HIV infection could interfere with HIV transcription, replication, and disease progression (38). At the same time, in MIP+TNF-IL-2-CD8+ T cell subsets, the nef gene-specific response was increased, and the pluripotent subsets of CD8 cells were increased (39). These data reveal that macrophages are closely associated with the progression of HIV infection.

### Interaction of Other Innate Immune Cells With HIV Infection

NKT cells play an immunity role by secreting many cytokines and chemokine and bridging innate immunity to adaptive immunity. NKT cells can be divided into type I and type II, according to the differences of T cell surface receptor (TCR) and antigen (40). TypeINKT cells can further be divided into three subtypes, NKT1, NKT2, and NKT17, which can enhance the ability to produce IFN-γand IL-21, thus activating cytotoxic T-lymphocyte more effectively (41). On the contrary, type II NKT cells can inhibit the function of CD8+T cells and weaken their cytotoxicity, thus reducing the killing ability of tumor cells and promoting tumor progression (42). In a cohort of 22 HIV patients with a mix of LTNPs, progressors, and asymptomatic HIV-infected individuals, researchers found a definite shift from NKT1 to NKT2 in HIV-infected individuals (43), suggesting that different NKT cells play a significant role in the progression of HIV infection. Besides, some studies found that increased levels of CD244 expressed on NKT cells were associated with HIV disease progression (44). Furthermore, a study (45) found that NKT cells secreted more IFN-γ, IL-2, and TNF-α in LTNPs compared to progressors, and these cytokines could significantly decrease the plasma viral load and maintain higher CD4+ T cell counts as well, suggesting that NKT cells in LTNPs secrete a large number of cytokines and might be one of many factors needed for HIV control.

In general, the translocation of microbial products from the gastrointestinal tract to the portal vein and systemic circulation is a major driver of pervasive chronic immune activation associated with HIV disease progression (46). Mucosal-associated invariant T (MAIT) cells are characterized by the combined expression of the semi-invariant T cell receptor (TCR) Vα7.2, the lectin receptor CD161, as well as IL-18R, and play an important role in antibacterial host defense of the gut (47). In a cohort study of patients with slow disease progression and ECs (48), MAIT cells were analyzed after *in vitro* stimulation with different cytokines and/or stationary Escherichia coli. CD 161(+) MAIT cells were reduced in blood and lymph nodes in all patient groups during HIV infection, including ECs. CD161+ MAIT cell numbers did not recover even after successful ART, and this deletion was associated with higher levels of MAIT cell activation. γδ T cells also play a dominant role in the intestinal mucosa, helping to maintain intestinal environmental stability and mucosal immunity, and express semi-constant TCR, which can be divided into two main subgroups, Vδ1 or Vδ2, according to gene differences (49). Vδ1+ cells are primarily located in intraepithelial lymphocytes (IELs) in the intestinal mucosa and help maintain epithelial function, while Vδ2+ cells mainly circulate in the blood (50). The frequency of Vδ1+ cells is increased in HIV-infected individuals, including ECs. Importantly, increased frequency of systemic pro-inflammatory Vδ1 + γδ T cells in ECs correlates with gut viral load. This study suggests that local viral replication in the gut in ECs disrupts the phenotype and function of Vδ1+ cells and thus may contribute to systemic immune activation and HIV disease progression (51).

For other innate immune cells, such as eosinophils and mast cells, the mechanisms of action in ECs of HIV infection have not been reported yet.

## Participation in the Innate Immune Pathway of HIV Infection Without Progression

**Figure 2:**
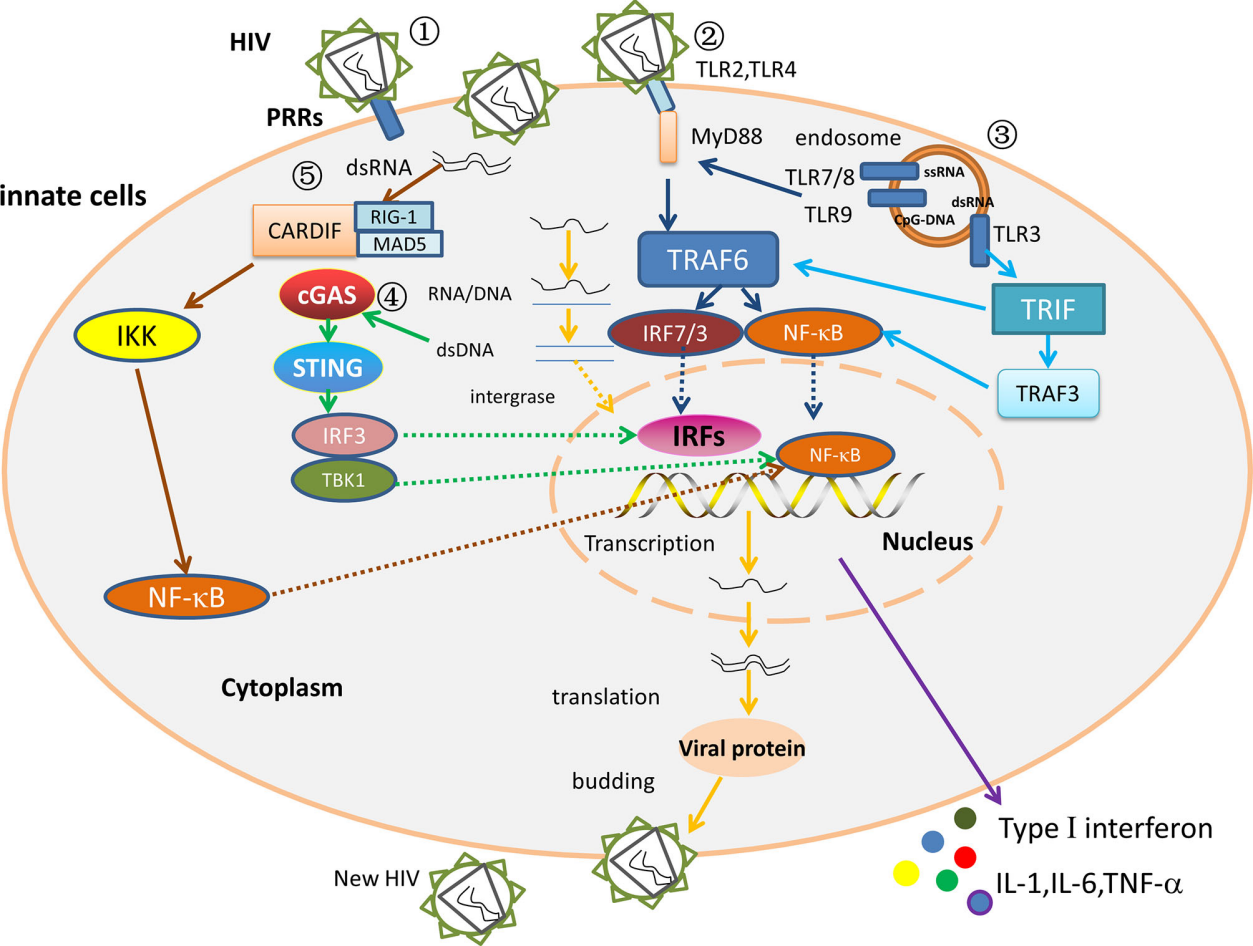
The interaction between the innate immune factors. ① The HIV replication cycle. After PRRs recognize and bind to HIV, the viral outer membrane fuses with the cell membrane, and the viral RNA enters the cytoplasm. Under the action of reverse transcriptase, viral RNA is reverse transcribed to produce RNA-DNA hybrid strands, which are subsequently transcribed to produce double-stranded DNA. The viral double-stranded DNA integrates into the pre-viral gene of host DNA, which is then latent or transcribed into mRNA, and then translated to viral protein and packaged into a complete virus. The mature progeny virus is released to produce new HIV by budding. ② MyD88 dependent pathway. In ECs/LTNPs, HIV is recognized by TLR7/8 and TLR9, and the activated TLRs stimulate the MyD88 signaling pathway, then activate TRAF 6 and recruit NF-κB and IRF7, and increase the production of type I interferons, TNF-α, IL-1, and IL-6, thereby controlling virus replication and delaying disease progression. ③ MyD88-independent pathway in ECs or LTNPs. The dsRNA binds TLR3, then stimulates the TRIF signal pathway, raises TRAF3/TRAF6, and recruits NF-κB and IRF3 to trigger type I interferons and TNF-α. ④ The DNA receptor signaling pathway in ECs/LTNPs. The dsDNA binds cGAS, stimulates STING signal pathway, recruits IRF3/TBK1, then activates NF-κB and IRFs, and triggers type I interferons, TNF-α, IL-1, and IL-6 production, thus slowing the disease progression. ⑤ The RNA receptors signaling pathways in HIV infection. The RIG-1/MDA5 recognizes dsRNA, binds Cardif in the mitochondria, raises IKK, and activates NF-κB to trigger type I interferons production. However, the role of RLRs signaling pathway in ECs remains unclear.

The innate immune signaling pathway mainly identifies invading viruses through pathogen-associated molecular patterns (PAMPs). The corresponding receptors are called Pattern recognition receptors (PRRs) (52), mainly including Toll-like receptors (TLRs), RIG-I-like receptors (RLRs), DNA receptors, and Nod-like receptors (NLRs) (53). The distribution of PRRs in various cells is different. For example, TLRs are distributed in various cell types and are expressed on the cell surface or in the endosome, and RLRs are cytoplasmic PRRs widely expressed in most cell types (54). NLRs are mainly expressed in the cytosol of various cell types, including epithelial, antigen-presenting cells, and adaptive immune cells (55). When these PRRs bind to their ligands, their conformation changes in such a way that they recruit specific cytoplasmic junction proteins, triggering a signaling cascade that causes type I interferons, pro-inflammatory cytokines, chemokine, and a series of antiviral factors are produced, which expressed products work together to inhibit viral replication, induce apoptosis of infected cells, and promote adaptive immunity to eliminate infected viruses (56). After HIV invasion in LTNPs/ECs, these receptors suppress viral infection by activating innate immunity and promoting the initiation of adaptive immunity. The interaction of innate immunity in ECs infected with HIV is depicted in **Figure 2**.

### Relationship Between Toll-Like Receptor (TLR) Signaling Pathways and HIV Infection Progression

Toll-like receptors (TLR) are a group of receptors that are closely related to innate immunity that play an essential role in maintaining immune system function through the recognition of pathogen-associated molecular patterns (PAMPs), mediating the secretion of host-related cytokines and the induction of the innate immune response (57, 58). According to the different intracellular locations of TLRs, they can be divided into two subfamilies, namely TLR1, TLR2, TLR4, TLR5, TLR6, and TLR10 located on the surface of the cell, and TLR3, TLR7, TLR8, and TLR9 located in intracellular organelle membranes, such as intracellular bodies, lysosomes and the endoplasmic reticulum. TLR signal transduction pathways mainly include MyD88-dependent pathways and MyD88-independent pathways. TLR1, 2, 5, 6, 7, 8, and 9 depend on MyD88, TLR3 is independent of MyD88, and TLR4 employs both of these signaling pathways (59, 60). In MyD88-dependent pathways, after TLRs combine with intracellular junction protein MyD88, downstream IRAK (IL-1 receptor-associated kinase) and TRAF (TNF receptor-associated factor) are successively involved in the signaling process, and finally activates the transcription factor NF-κB or Mitogen-activated protein kinase (MAPK), and then activate interferon regulatory factors (IRFs), which generate a variety of pro-inflammatory factors, causing inflammation (61). Regarding the MyD88-independent pathways, TLR3 and TLR4 recognize HIV, bind to TRIF (TIR domain-containing adaptor inducing interferon β), recruit downstream TRAF3 and TRAF6, and then activate NF-κB and IRF3, respectively, induce the expression of inflammatory factors and typeIinterferons, which have antiviral effects (62).

TLRs can activate MyD88 and mediate NF-κB signaling pathway activation. After performing flow cytometer analysis on 48 HIV-infected and 21 healthy people, some researchers observed that (63) HIV gp120 could induce increased TLR2 expression in HIV-infected monocytes, and activation of TLR2 led to increased viral replication and TNF-α production. Meanwhile, the researchers also noticed that TLR2 could affect the phenotype by activating resting and memory CD4+ T cells, increasing sensitivity to the HIV X4 and R5 subtypes of the virus. Therefore, it was speculated that derivatives of HIV induced the expression of TLR2 in resting CD4+T cells, thus inducing the acquisition of resting and memory CD4+T cell effector phenotypes and leading to the acceleration of viral replication, immune disorders, and the progression of AIDS. These results suggest that TLR2 plays an important role in persistent immune response and virus replication in HIV infection. It has been reported (64) that, in HIV infection, the TLR4-mediated signaling pathway promotes the viral transcription process, but this signaling pathway is an extremely complex cascade process, and the related pathogenesis has not been fully elucidated. Interestingly, other results (65) showed that a TLR4 gene polymorphism (896A/G) could affect disease progression after HIV infection, showing that the activity of the mutant TLR4 was weakened and disease progression after HIV infection could be delayed.

Intracellular TLRs such as TLR3, TLR7, TLR8, and TLR9 play key roles in detecting viral nucleic acids in human cells. Intracellular TLRs and virus PAMPs stimulate NF-κB and IFN response elements, and other transcription factors stimulate antiviral factors such as type I interferons, pro-inflammatory factors, and chemokine (66). When TLR3, TLR7, TLR8, and TLR9 agonists were added before HIV-1 infection, they significantly reduced peripheral blood mononuclear cell infection. Interestingly, the addition of TLR8 and TLR9 agonists 48 h and 72 h after HIV-1 infection, respectively, were highly effective in preventing HIV replication. Analysis of the induction of antiviral genes after TLR activation by agonists showed that all agonists induced the expression of type I interferons and interferon-stimulating genes, although the level depended on the agonist used. These findings suggest that endoplasmic reticulum TLR agonists can induce the expression of anti-HIV molecules through TLR-mediated and non-TLR-mediated mechanisms (57).

On the other hand, it has been confirmed that the activation of TLR7 after HIV infection leads to inflammation, a reduction in lymphocytes, and the destruction of lymphoid tissues (67). In addition, uracil-rich RNA sequences in HIV nucleic acids could act as TLR recognition ligands and are recognized by TLR7/8 in monocytes and dendritic cells, thereby initiating TLR signaling dependent on MyD88 to induce B7-H1 expression (68). Being compared to progressors, LTNPs produce higher levels of IFN-α and apoptotic pathway ligands by increasing expression of IRF7 and MyD88 in TLR7/8, suggesting that the TLR7/8-MyD88 activation pathway plays a role in disease progression (69). Besides, by comparing LTNPs with slow progressors, some researchers found that the expression of TLR7/8 mRNA and TNF-α secretion in peripheral blood monocytes decreased with disease progression, indicating that the triggering of TLR7 and TLR8 can inhibit HIV replication in monocytes (70). Furthermore, compared with the viremia group, the overall up-regulation of TLR signaling pathways in LTNPs is manifested by increased expression of TLR 7/8 at the transcriptome level, followed by increased expression of genes encoding MAPK, NF-κB, and IRF signaling cascades, as well as cytokines, which further confirm that the activation of TLR7/8 can delay the progression of HIV disease (71).

In addition, evidence suggests that single-nucleotide polymorphisms (SNPs) in TLR9 genes are associated with accelerated HIV disease progression. Some researchers analyzed 28 SNPs in TLRs in a Swiss HIV-positive patient cohort and found that two SNPs in TLR9 (1635A/G and +1174G/A) correlated rapidly progressing patients’ phenotype, indicating that the rapid progression of HIV infection was correlated with TLR9 polymorphism (72). Moreover, it was confirmed in another study performed in India that TLR7 SNPs may be one of the factors related to HIV susceptibility, and the 1635A/G allele of TLR9 might play a role in the progression of HIV disease (73). Additionally, some studies conducted whole-exome sequencing of 7 LTNPs and 4 ECs and found that, in the LTNPs, some rare mutations were found in genes involved in innate immune sensing, CD4-dependent infection, HIV trafficking, and HIV transcription. Compared to a matched control group, variations in the TLR and NOD2 pathways in the slow HIV progressors showed lower pro-inflammatory mediators, which might help understand the pathogenesis of HIV and the slow progression phenotype (74).

Furthermore, the TLR signaling pathway was down-regulated in the disease progression group with lower MAPK and NF-κB activation, while the opposite was seen in LTNPs (71). The decrease in cytokine gene expression observed in the TLR signaling pathway relative to LTNP was further demonstrated by down-regulation of the cytokine-cytokine receptor interaction pathway in the viremia group. One of the most significant changes in this pathway was the effective and systematic down-regulation of genes encoding pro-inflammatory cytokines, including IL-1B, IL-6, IL-8, TNF-α, in the viremia group. Interestingly, the core-enriched gene IL-15 shares many activities with IL-2 and is down-regulated in patients with viremia, and was also found to be expressed at significantly higher levels in LTNPs monocytes than in people with HIV progression (75). Finally, a study has shown that at least one host genetic factor (HLA, chemokine receptors is associated with slow HIV progression (76).

### Interactions Between Intracellular Nucleic Acid Recognition Signaling Pathways and HIV Infection

The etiologic factor of AIDS is HIV. After infection of the body, HIV activates a series of nucleic acid receptors in cells, including RLRs, and AIM2-like receptors (AIM2 and IFI16), as well as the newly discovered cyclic GMP/AMP synthase (cGAS) (77). Previous studies have found that, in the course of the HIV replication cycle, RNA, single-stranded DNA, hybrid RNA-DNA, and double-stranded DNA are produced; these nucleic acids are all important PAMPs for the body to recognize HIV. The cGAS-STING signaling pathway has been confirmed to have an antiviral effect. As a newly discovered DNA membrane receptor, cGAS can recognize the double-stranded DNA of the HIV precursor. It produces endogenous cGAMP, which activates STING. STING is a key protein in the DNA receptor pathway and plays an important role in innate and adaptive immune initiation (78). Type I interferons have been found to play a pathogenic role in the pathogenesis of HIV so that reduced IFN responses may be the basis of the LTNP phenotype. A recent study (79) performed Sanger sequencing on a Danish HIV group and found that LTNPs had lower, innate immune responses for DNA and HIV expression and reduced CD4+ T proliferation-dependent stimulation than non-control subjects receiving antiretroviral therapy. These findings show that the homozygous HAQ STING mutation contributes to reduced inhibition of CD4+ T cell proliferation and reduced immune responses to DNA and HIV, leading to reduced IFN production. Thus, the HAQ/HAQ TMEM173 genotype might help to slow down disease progression in LTNPs. In addition, an analysis of 46 ECs, 7 LTNPs, and 50 healthy controls of HIV infection found that endogenous levels of IFN-λ1, IFN-β, and RANTES were higher in HIV-infected patients than in the control group at baseline. Gene induction was induced in both HIV-infected and healthy controls after DNA transfection; however, the induction was significantly lower in HIV patients than in uninfected controls. Therefore, ECs have a lower level of the innate immune response through the cytoplasmic DNA detection system, which might be caused by continuous immune activation (80). Furthermore, some researchers have illustrated that STING activators could activate innate immunity, induce the generation of antigen-presenting cells and stimulate inflammatory cytokine expression to promote the activation and recruitment of T cells and slow down disease progression (81).

RLRs are a class of important pattern recognition receptors for viruses, including RIG-1, MDA5, and LPG2, which can play an important role in antiviral infection by inducing the RLR signaling pathway (82). After RLRs recognize the double-stranded RNA, the Cardif connector protein of the mitochondria is connected to recruit I κB kinase (IKK), activate NF-κB and IRF-3, and induce the secretion of type I interferons to exert antiviral effect (83). The most obvious characteristics of the acute phase are the increased expression of hundreds of genes involved in immune activation, innate immune defenses (such as RIG-1, MDA-5, TLR7 and TLR8, PKR, DDX3), adaptive immunity, and the pro-apoptotic ligand pathway (ApoE-Fas), which are associated with HIV infection (84). Some researchers (85) have used real-time RT-PCR to detect the expression of pattern recognition receptor (PRR) mRNA in the female reproductive tract and found that exposure to HIV IIIB but not exposure to Bal led to the selective up-regulation of NOD2 and MDA5. It was further found that (86) the production of HIV in FTSJ3 knockdown cells showed a decrease in 2’-O-methylation and triggered the expression of type 1 interferon in human dendritic cells through the RNA sensor MDA5; the induction of IFN-α and IFN-β led to a decrease in HIV expression. In addition, studies have shown (87) that DDX3 RNA helicase is essential for the initiation of translation of HIV mRNA and replication of the virus. DDX3 can also detect virus inactivation RNA, but HIV actively inhibits DDX3-mediated signal transduction, thereby inhibiting antiviral immunity.

### The NOD-Like Receptor Signaling Pathway and HIV Infection

NOD-like receptors are crucial PRRs of the innate immune system, including NLRP1, NLPR3, and NLRC4. HIV infection activates a NOD-like receptor signaling pathway. NOD receptors recognize ligands and activate receptor-interacting serine-threonine kinase (RICK), and then activate NF-κB and Caspase-1, respectively, and accordingly induce the expression of inflammatory cytokines and cytokines IL-1 and IL-18 (88). Some results have indicated that the cGAS-STING pathway can regulate NLRP3 through potassium ions. Additionally, NLRP3-induced pyroptosis leads to the mass death of CD4+T cells and aggravates a severe reaction by releasing inflammatory factors and cell contents (89). Furthermore, it has been found (90) that pyroptosis caused by caspase-1 activation in the NOD-like receptor signaling pathway might also affect the number of CD4+ T cells. However, the specific mechanisms of the key factors of the cGAS-STING-NLRP3 signaling axis and caspase-1 activity in LTNPs are not yet clear.

### Relationship Between Other PRR Receptors and HIV Infection Progression

Dendritic cells are among the earliest receptors of HIV after mucosal exposure and can bind the virus *via* C-lectin receptors (CLRs). It has been confirmed that CLRs play a significant role in transferring HIV to T cells (91). External cervical biopsy specimens from women with different HIV risks showed that the expression density of CLR was significantly higher in high-risk women than that in low-risk women, suggesting that CLRs might be associated with HIV infection (92). For other PRRs, such as scavenger receptors and complement receptors, no studies have reported a related mechanism of action in the non-progression of HIV infection.

## The Relationship Between Soluble Factors, Co-Receptors, and Disease Progression

Cytokines and chemokine perform a vital function in all stages of the life cycle of HIV, from the binding of the HIV envelope to cell surface receptors to the budding stage (93). Some cytokines might limit virus transmission, while others might promote virus transmission (94). *In vivo* studies have shown that the levels of IP-10, MCP-1, MIP-1α, and IL-21 were the same in the serum of ECs and uninfected individuals, but the TGF-β level was higher. At the same time, compared with HIV-infected patients who could not control viral load naturally, the levels of IP-10, MCP-1, and TGF-β were lower, while that of MIP-1α was higher (34, 95). A study of 24 LTNPs found that IL-8 levels were significantly higher in progressors than in LTNPs, but there was no difference between the LTNP subgroups. CD4+ T cell counts were negatively correlated with IL-8 levels and positively correlated with CD8+ T cells (96).

On the other hand, after describing the TH1, TH2, and TH17 cell profiles, researchers (97) found that the production of IL-6, IL-10 (TH2), TNF-α and IFN-γ (TH1) was observed in ECs, and these patients presented a non-polarised cytokine production profile. IL-2 and IL-4 were not detected, possibly due to a 13-year- duration of infection, as these cytokines are part of a characteristic pattern of early infection. Besides, higher levels of IL-17 were observed, confirming the hypothesis of higher levels of IL-17 production in ECs with viral road < 50 copies/mL. Moreover, some studies have shown that (96, 98), with a decrease in the number of CD4+ T cells, compared to the low level of IP-10 in LTNP plasma, the IFN-γ level was higher in the control group. At the same time, the study also found that, compared with LTNPs, there were more specific CD8+ T cells in progressors. Moreover, the levels of IP-10 were associated with viremia, and the EC subgroup had nearly identical levels of chemokine as in healthy individuals and progressors with a suppressed viral load. Meanwhile, CD4+ T cell counts, CD4+ T cell percentages, and CD4/CD8 ratios were negatively correlated with IP-10. However, no correlation was found between the plasma levels of IL-8 and IP-10, which suggests that IL-8 and IP-10 are related to disease progression after HIV infection.

Moreover, in the early stage of HIV infection, gp120 membrane proteins are required to interact with CD4 and CC family chemokine receptor 5 (CCR5) and can only enter the cell after binding; this is called the M-addicted stage or R stage. CCR5 Δ32 is a common mutation in CCR5 that can lead to a change in protein function and the loss of HIV co-receptor function. Several studies have shown that the CCR5Δ32 gene locus can effectively control infection (99–102). In addition, some research results (103) indicate that the expression of CXCR2, CXCR4, and CCR5 are significantly changed in the peripheral blood mDC and pDC of HIV+ progressors. In contrast, the expression of chemokine receptors and the number of CD16+ DC in HIV+ progressors were not different from those in healthy adult volunteers but were altered in HCV+/HIV- and LTNPs. These results suggest that the expression of chemokine receptors might be associated with HIV disease progression.

## Relationship Between Other Factors and the Progression of HIV Infection

### Relationship Between Human Leukocyte Antigen (HLA) and the Progression of HIV Infection

HLA is a complex genetic polymorphism system, which controls the immune response and immune regulation and determines the histocompatibility of the body. Class I HLA molecules play an important role in regulating innate immune response (104). Infectious and inflammatory diseases repeatedly show strong genetic associations in the major histocompatibility complex (MHC). However, the basis of these associations remains unclear. To determine the influence of host genes on the outcome of chronic viral infection, Bruce Walker’s group recruited a multi-ethnic cohort of HIV-1 controllers and progressors to conduct a genome-wide association study (GWAS) in the absence of antiviral therapy, showing that >300 genome-wide significant SNPs were found in MHC, but not elsewhere. HLA - B peptides based on specific amino acids in the groove, as well as independent HLA- C effects, explain the SNP association and reconcile protective and risk HLA alleles, which indicate that the nature of HLA-viral peptide interactions is the major genetic factor modulating durable control of HIV in ECs (105). There is no doubt that this is a landmark study in the field. On the other hand, different HLA alleles have different effects on disease progression, and the combination of HLA alleles also strongly influences the LTNPs (106). For example, HLA-A (*) 30-B (∗) 13-C (∗) 06 and B (∗) 67 are associated with the long-term non-progression of HIV-1 infection (107). Santors’ study of 24 LTNPs found that IL-8 chemokines were significantly higher in progressors than in LTNPs, but there was no difference between LTNP subgroups. In the ECs subgroup, 80% of patients had at least one HLA-B allele, which was previously considered to have a potential protective effect on the progression of AIDS, but no association between the HLA-B allele and HIV-1 was observed (96). Therefore, the mechanism between HLA-associated host genetic factors and the natural control of HIV in the LTNPs/ECs population still needs further study.

### The Relationship of ART, Functional Cure, Vaccine Development With ECs

The most striking feature of ECs is the complete suppression of viremia to undetectable levels, which can be achieved with ART, and both can effectively prolong the survival of AIDS patients and reduce AIDS-related deaths. ART inhibits viral replication for decades and rebuilds the immune system, mainly by increasing circulating CD4+T lymphocytes (108). ART also causes some innate immune-related changes. ART control of HIV-1 viremia leads to functional recovery of NK cells, such as mature phenotypic NK cells. Although ART helps restore the subset allocation of NK cells, there is still a defect in NK cells to produce IFN-γ and cytokines (109). ART also facilitates DCs to regain the ability to properly mature and secrete cytokines required to activate NK cells and the production of IFN-γ, such as IL-12, IL-15, and IL-18 (110). Besides, although DCs in ART-treated HIV patients produce a small amount of IL-12, they could not prevent DCs maturation or activation of CD8+ and CD4+T cells (111, 112), suggesting that DCs in ART-treated HIV patients maintain their complete function. Additionally, the activation level of γδ T cells in HIV-infected patients was significantly enhanced, and ART did not reduce the activation level of γδ T cells in HIV-infected patients (113). These are similar to how ECs control HIV replication in innate immunity. Some unique and desirable features of ECs are absent in non-controllers on ART, so it is clear that, in contrast to EC, suppression of viremia in ART-treated patients requires lifelong medication. It has also been demonstrated that ECs have smaller HIV reservoirs, and recovering the virus from EC seems to be more difficult than progressors treated with ART (114, 115). On the other hand, in an early-treated case of congenital HIV infection, using a full dose of immediate ART after birth, viral replication is continually suppressed despite a long interruption of treatment (116). This study suggests that early ART introduction in infected people who cannot spontaneously control infection may induce a similar state of HIV replication control as ECs.

There seems to be an excellent mode of functional cure in ECs, in which the virus, though not eliminated, is maximally suppressed and the patient’s defense mechanisms maintain the integrity of the immune system relatively intact, without the need for lifelong drug therapy. Compared with ECs, HLA-B57 or B27, the most recognized genetic elements associated with HIV control, were not abundant in the acute treatment group but abundant in HLA-B35, associated with rapid disease progression. Surprisingly, HIV DNA banks were similar in size and distribution to ECs in patients who became controllers after treatment (117). Undoubtedly, the insights gained from the innate immune mechanisms of ECs discussed above will provide significant support for achieving a functional cure for AIDS.

In addition, another goal of strengthening ECs research is to contribute to the development of an effective HIV vaccine. In fact, over the past two decades, several important immune-related factors found from ECs, have been used to improve vaccine effectiveness. However, to develop a more effective vaccine, there is an urgent need for more definitive immunization measures to predict vaccine effectiveness in HIV vaccine development. Therefore, further study of ECs, an innate immune mechanism that can control the progression of HIV disease, will help provide new targets for the development of HIV vaccine to control the epidemic of AIDS effectively.

### The Relationship of Chronic Immune Activation With ECs

It is well known that AIDS is a chronic disease, and chronic immune activation is one of the pathological characteristics of HIV infection. The phenomenon of chronic immune activation in AIDS was first proposed by As-Cher and Sheppard (118). After HIV infection, the continuous stimulation of HIV antigen puts the immune system in an abnormally chronic activated state, an important feature of AIDS and the driving force of HIV-related immune deficiency (119). The role of chronic immune activation in non-AIDS-related complications has been widely accepted, such as malignant tumors, cardiovascular disease, threatening patients’ life quality and even life span. It is now clear that even undetectable plasma viremia is insufficient to prevent such complications. In ECs, although standard assays did not detect plasma viremia, there was persistent replication of the virus, which may play an important role in the pathogenesis-related to chronic immune activation (120). This idea is further supported by evidence that HIV can evolve in ECs, suggesting that sustained exposure to low levels of viral product release, the immune system may trigger sustained immune activation and a chronic inflammatory state in ECs (121).

On the other hand, higher comorbidity rates were still found in ECs compared to uninfected controls due to the detrimental effects of ongoing inflammation and immune activation. Levels of innate immune activation, such as CD16 and IL-6 produced by monocytes and IFN-α produced by dendritic cells, are elevated and associated with increased morbidity in treated and untreated patients with chronic infection (122). In addition, innate immune activation markers, such as CD163, monocyte activation markers, have increased coronary heart disease incidence. Accelerated atherosclerosis is also found in ECs and is associated with the inflammatory marker C-reactive protein (123). These findings suggest that although ECs can naturally control the virus below detection levels, there is still a significant level of immune activation that may lead to various forms of end-organ damage.

## Discussion

In response to HIV infection, innate immunity plays a two-fold role. Not only it directly inactivates or attenuates invading pathogens, but also it can act as a bridge that mediates the activation of the adaptive immune system. Innate immune cells, such as dendritic cells, macrophages, and NK cells, can distinguish self from non-self through PRRs interacting with PAMPs, and then secrete cytokines and chemokine to exert antiviral effects. In ECs/LTNPs, the disease progression is affected by activating different innate immune components after HIV invaded the host. On the one hand, ECs or LTNPs can control the replication of the virus by an increasing number and activity of some innate immune cells, including DCs, NK cells, NKT cells, and macrophages. At the same time, the secretion of cytokines such as IFN-α, IL-2, IL-4, IFN-γ, and TNF-α, are beneficial to delay the disease progression of AIDS. On the other hand, activation of PRR pathways such as TLR2 and TLR4 may lead to inflammation, promoting HIV replication and accelerating the progression of AIDS, while TLR7 and TLR8 may increase the expression of NF-κB, inhibiting HIV replication and delaying the progression of AIDS in ECs/LTNPs. Furthermore, the polymorphism of TLR7 and TLR9 SNPs are associated with HIV susceptibility, and some mutations may delay the development of AIDS in ECs or LTNPs. In addition, in ECs or LTNPs, the cGAS-STING signaling pathway is activated, which can induce the production of inflammatory cytokines and delay the progression of the disease. Nevertheless, the role of RLRs in ECs/LTNPs is still unclear. Although plasma viremia was not detected in ECs, there was persistent replication of the HIV and sustained exposure to low levels of viral product release, and the immune system may trigger sustained immune activation and a chronic inflammatory state. Thus, higher comorbidity rates are still found in ECs due to the detrimental effects of ongoing inflammation and immune activation.

In our opinion, there is a double-edged sword of innate immunity for HIV infection. In the early stages of HIV infection, a high level of immune activation may be beneficial in controlling the development of HIV. However, continuous immune activation can easily lead to immune system disorders, resulting in chronic inflammation and other diseases. Therefore, low levels of immune activation may be more conducive to controlling HIV disease progression in the later stages of HIV infection. Early initiation of ART can inhibit HIV replication for a long time, even if treatment is discontinued for a long time. Therefore, is it possible to mitigate the adverse effects of chronic immune activation with the early introduction of ART in specific populations such as ECs, worthy of further investigation.

There is no doubt that the study of ECs of HIV infection has always been a hot topic to realize the functional cure of AIDS. Elite control is almost equivalent to a functional cure. Although the virus is not completely cleared, the body’s immune function is normal for several years even without antiviral treatment, and the virus cannot be detected in the blood with conventional methods, which is the most ambitious goal in the field of HIV research. Because there are no cases of HIV infection cured by drug treatment, the ECs are the only research model that can provide ideas for new treatment and vaccines. We can try to find this natural suppression principle from ECs, and attempt to crack the uniqueness of this group. Fortunately, current research on innate immune mechanisms in these populations is a good springboard that may inform vaccine design and identify potential new antiviral targets to help us create a definitive and effective AIDS vaccine.

To sum up, in natural ECs of HIV infection, the host controls the progression of the disease by activating a variety of innate immune-related immune cells, cytokines, and signaling pathways or by activating these pathways at different levels. At present, great progress has been made in the study of innate immune mechanisms in the disease progression of ECs with HIV infection, which provides new targets and theoretical references for the functional cure, prevention, and control of AIDS and the development of vaccines. Nevertheless, the key regulatory mechanisms of disease progression and innate immunity in natural elite controllers of HIV infection remain unclear due to the extremely low proportion and difficulty in the recruitment of LTNPs and ECs of HIV infection and the immune system’s complexity. Therefore, further research into the mechanism of action by which innate immunity controls disease progression in ECs is conducive to further clarifying the mechanism of non-progression and ultimately contributing to the effective prevention, treatment, and control of the global HIV epidemic.

## Author Contributions

YS, JS, and RC have contributed equally to this work and share the first authorship. WW, ZY, XC, and XW discussed the concepts of the manuscript. HL, LY, and JJ approved the version to be submitted. All authors contributed to the article and approved the submitted version.

## Funding

The study was supported by National Natural Science Foundation of China [NSFC, 82103898, 81971935, 81960602, 31860040], Guangxi Bagui Scholar (to JJ), Guangxi Medical University Training Program for Distinguished Young Scholars (to JJ), Guangxi Science Fund for Distinguished Young Scholars (2018GXNSFFA281001).

## Conflict of Interest

The authors declare that the research was conducted in the absence of any commercial or financial relationships that could be construed as a potential conflict of interest.

## Publisher’s Note

All claims expressed in this article are solely those of the authors and do not necessarily represent those of their affiliated organizations, or those of the publisher, the editors and the reviewers. Any product that may be evaluated in this article, or claim that may be made by its manufacturer, is not guaranteed or endorsed by the publisher.
